# Sustainable *Cannabis* Nutrition: Elevated root-zone phosphorus significantly increases leachate P and does not improve yield or quality

**DOI:** 10.3389/fpls.2022.1015652

**Published:** 2022-11-17

**Authors:** F. Mitchell Westmoreland, Bruce Bugbee

**Affiliations:** Crop Physiology Laboratory, Department of Plants, Soils and Climate, Utah State University, Logan, UT, United States

**Keywords:** *Cannabis*, plant nutrition, phosphorus, phosphorus use efficiency, phosphorus partitioning, controlled environment agriculture

## Abstract

Phosphorus (P) is an essential but often over-applied nutrient in agricultural systems. Because of its detrimental environmental effects, P fertilization is well studied in crop production. Controlled environment agriculture allows for precise control of root-zone P and has the potential to improve sustainability over field agriculture. Medical *Cannabis* is uniquely cultivated for the unfertilized female inflorescence and mineral nutrition can affect the yield and chemical composition of these flowers. P typically accumulates in seeds, but its partitioning in unfertilized *Cannabis* flowers is not well studied. Here we report the effect of increasing P (25, 50, and 75 mg P per L) in continuous liquid fertilizer on flower yield, cannabinoid concentration, leachate P, nutrient partitioning, and phosphorus use efficiency (PUE) of a high-CBD *Cannabis* variety. There was no significant effect of P concentration on flower yield or cannabinoid concentration, but there were significant differences in leachate P, nutrient partitioning, and PUE. Leachate P increased 12-fold in response to the 3-fold increase in P input. The P concentration in the unfertilized flowers increased to more than 1%, but this did not increase yield or quality. The fraction of P in the flowers increased from 25 to 65% and PUE increased from 31 to 80% as the as the P input decreased from 75 to 25 mg per L. Avoiding excessive P fertilization can decrease the environmental impact of *Cannabis* cultivation.

## Introduction

There is a growing interest in controlled environment agriculture (CEA) to supply plant derived food and medicine to a growing world population (Kalantari et al., 2017). CEA has the potential to improve crop productivity and limit the environmental impact of field agriculture by precisely delivering the necessary conditions for optimal growth. High-quality fertilizer is essential in high-input CEA systems, but excess fertilization depletes reserves of finite resources and pollutes critical ecosystems (Parry, 1998). CEA provides a unique opportunity for a more sustainable approach to agriculture.

Phosphorus (P) typically occurs in high concentrations in fruits and seeds, and ensuring adequate P throughout the lifecycle can decrease time to flower and increase flower number in some species (Ma et al., 2002; Malhotra et al., 2018). However, excessive P in the root-zone can interfere with uptake of other essential nutrients (Parry and Bugbee, 2017) and have a detrimental environmental impact (Parry, 1998). The P concentration in continuous liquid fertilizer is considered adequate at 10 to 20 mg per L for most species (Bugbee, 2004) but lower concentrations may be adequate for some floriculture species (Henry et al., 2017; Henry et al., 2019). The P concentration in liquid feed is often reduced to less than 10 mg per L to reduce stem elongation in bedding plants (Nelson et al., 2012) but this concentration may not be adequate for all species.

Medical *Cannabis* has two broad growth stages – vegetative and reproductive – that may have different P demands for optimal growth and development. Few studies have evaluated the effect of P supply during vegetative growth of *Cannabis*, but data from other crops suggest a potential difference in P demand based on growth stage (Wang et al., 2016). Rapid P uptake in wheat occurs early in the lifecycle and declines to near zero after anthesis (Römer and Schilling, 1986), while rice can accumulate as much as 40% of the total plant P after anthesis (Rose et al., 2010). Shiponi and Bernstein (2021a) found that 30 mg P per L was necessary for maximum dry mass after four weeks of vegetative growth in two high-THC *Cannabis* cultivars. In contrast, Cockson et al. (2020) found no increase in dry weight above 11 mg per L after eight weeks of vegetative growth in a high-CBD cultivar, despite P concentration in leaves nearly doubling as the P input increased from 11 to 30 mg per L.

The potential benefit of extremely high P in *Cannabis* is thought to occur during flowering. It is a common commercial practice to apply rates exceeding 100 mg per L, but few studies have rigorously evaluated the effect of this high rate of P on yield and quality. Medical *Cannabis* may have a uniquely high uptake of P during reproductive growth because the developing inflorescences can have three-times the P concentration of the leaves (Bernstein et al., 2019; Shiponi and Bernstein, 2021b) and can be up to 65% of the dry mass at harvest (Westmoreland et al., 2021), but uptake does not imply a requirement for growth. A high P concentration of the inflorescences is not known to have a beneficial role in metabolic pathways, and the P is likely in storage forms. Nutrient uptake in excess of metabolic requirements is called “luxury uptake”. This excess uptake of P is common in algae (Powell et al., 2008) and crop plants (Kvakić et al., 2020).

In a comprehensive study with *Cannabis*, (Bernstein et al., 2019) found that supplementing constant liquid fertilization of 17 mg P per L with additional P from 2 g of superphosphate ([Ca(H_2_PO_4_)_2_]; 0.5 g P) per pot at three-week intervals did not increase flower yield or the concentration of three major cannabinoids (CBD, CBG, and CBN) in flowers compared to the control. Cockson et al. (2020) reported a significant increase in total above ground biomass up to 23 mg per L, but there was no increase in flower yield above 11 mg per L. Cannabinoid concentration was higher under P-deficient conditions, but there was no effect of P on cannabinoids above 11 mg per L (Cockson et al., 2020). In a similar study, there was no effect of P on flower yield from 15 to 180 mg per L (Veazie et al., 2021). Bevan et al. (2021) investigated the interactive effect of N, P and K on flower yield and quality using a central composite design in liquid hydroponics. They grew plants at P concentrations from 20 to 100 mg per L and predicted the optimal P concentration to be 59 mg per L (Bevan et al., 2021), but this study did not have the statistical power of other studies. Caplan et al. (2017a); Caplan et al. (2017b) investigated five rates of an organic fertilizer during the vegetative and flowering stage of *Cannabis*, but the concentrations of multiple nutrients changed as the rates increased so it is difficult to draw conclusions from this data.

P accumulates in high concentrations in the flowers of many species (Ignatieff, 1936; Li-xiang and Dan, 2014; Kim and Li, 2016), and has been shown to accumulate in the inflorescences of *Cannabis* (Shiponi and Bernstein, 2021b). In *Gladiolus*, P concentration was 45% higher in the petals and nearly 3 times higher in the ovaries than in the leaves (Ignatieff, 1936). In Lantana (*Lantana camara* ‘New Gold’), P concentration was 32 to 100% higher in the flowers than shoots and roots (Kim and Li, 2016). P concentration in the flowers of balloon flower (*Platycodon grandiflorum)* was two to three times higher than the leaves (Li-xiang and Dan, 2014). Shiponi and Bernstein (2021b) found that P concentration was 4 to 5 times higher in the flowers than in the leaves. P concentration in the flowers increased to more than 1% as P input increased to 30 mg per L, but there was no further increase as P input increased to 90 mg per L (Shiponi and Bernstein, 2021b).

Many plants have evolved to have high levels of storage P in their seeds to promote growth immediately after germination. This high P in seeds is often translocated from leaves to seeds during reproductive growth (Powers et al., 2020). In rice, 20% of the P in the grains at harvest was remobilized from vegetative tissue during grain fill (Julia et al., 2016). In vegetative tissue, P is typically stored as inorganic P in the vacuoles, while P in seeds is typically stored as phytic acid (PA) (Yang et al., 2017). PA is a myo-inositol phosphate that can account for nearly 4% of the dry weight of a seed in some species (Lott et al., 2000). Seeds of *Cannabis* have been shown to accumulate up to 0.7% P and 1.74% PA (Lott et al., 2000).

Medical *Cannabis* uniquely requires that the female flowers remain unfertilized. Biddulph and Brown (1945) demonstrated that P accumulation is most rapid in meristematic tissue of cotton flowers. After fertilization, genesis of new floral meristematic tissue typically stops (Machida et al., 2013), but unfertilized *Cannabis* flowers continue to grow and could therefore accumulate unnecessary P in floral meristematic tissue.

P is often limiting in biological systems. Unlike C, N and S, the natural cycling of P is slow and limited by the weathering of P rich minerals, which occurs more slowly than biological N fixation or atmospheric S deposition (Smil, 2000). Anthropogenic cycling of P occurs more rapidly, and is mainly driven by inorganic P-rich fertilization (Smil, 2000; Tirado and Allsopp, 2012; Cong et al., 2020). P is frequently over-applied in agriculture and is a major contributor to eutrophication (Daniel et al., 1998; Conley et al., 2009). Furthermore, supply of P is finite (Tirado and Allsopp, 2012; Schneider et al., 2019), which makes efficient P fertilization critical to our long-term food supply (Cordell and White, 2013; Scholz et al., 2013).

P moves into aquatic ecosystems from field environments through soil erosion and surface runoff (Alewell et al., 2020). This is accentuated by over-application of P coupled with irrigation or precipitation events that overload the water holding capacity of the soil. In controlled environments, P losses can be minimized by reducing the leaching fraction (Krofft et al., 2020), recycling leached irrigation water (Bar-Yosef, 2008), or using media amendments such as dolomitic lime to capture excess P before it leaches from the pot (Shreckhise et al., 2019).

Our objective was two-fold: 1) to investigate the effect of P concentration (25, 50 and 75 mg per L) in continuous liquid feed on yield and cannabinoid concentration (quality) of a high-CBD *Cannabis* cultivar during the flowering stage and 2) quantify the effect of increasing P on nutrient partitioning, leachate P and P use efficiency (PUE). We hypothesized that there would be no difference in yield or quality among treatments, but that there would be significant differences in leachate P, phosphorus partitioning and PUE.

## Materials and methods

### Plant material

Fifty cuttings of the medical hemp cultivar ‘Trump’ were harvested from the same mother plant and propagated in 3” deep cells filled with a 1:1 mix of peat:vermiculite that was pH adjusted to 5.8 using hydrated lime (Ca(OH)_2_). We used the medical hemp cultivar ‘Trump (T1)’ because it has high cannabinoids and a compact growth habit that is conducive to growth chamber studies where space is limited. After two weeks in propagation, 18 rooted cuttings were selected for uniformity and transplanted into 6.7 L plastic containers (#2 nursery pots) that were filled with a soilless mix of 6:1:1 peat moss, vermiculite, and rice hulls and randomly assigned to six groups. Each group consisted of three plants in three pots that shared a common leachate collection tray. Each group was randomly assigned to a P treatment (25, 50 or 75 mg per L) and placed in one of six leachate collection trays within an environmentally controlled growth chamber. Each P treatment was replicated twice (n=2). Plants were pinched to four nodes upon being moved into the growth chamber and grown under a vegetative photoperiod (18/6 hr day/night). After seven days of vegetative growth, the photoperiod was switched to an inductive photoperiod to promote reproductive growth (12/12 hr day/night). Plants were grown under an inductive photoperiod for 56 days (8 weeks). At harvest, the canopy area for each group was measured and used for yield and leached P calculations.

### Environmental conditions

The extended photosynthetic photon flux density (ePPFD; 400 to 750 nm) was 600 ± 30 µmol m^-2^ s^-1^ during vegetative growth (18 hr/6 hr light/dark; DLI: 38.9 mol m^-2^ d^-1^) and 900 ± 50 µmol m^-2^ s^-1^ during reproductive growth (12 hr/12 hr light/dark; DLI: 38.9 mol m^-2^ d^-1^) from white+red LEDs (BIOS Lighting Inc., Icarus Vi) (Kusuma et al., 2021). The fraction of far-red photons (700 to 750 nm) was 1.5%. Because the far-red fraction was low, the classic PPFD and the ePPFD were within 1.5% of each other (Zhen et al., 2021). Lights were dimmed as plants grew to keep a constant PPFD at canopy height throughout each growth phase. Temperature was 25.9 ± 0.4/23.3 ± 0.2 °C (day/night) measured with a shielded fan-aspirated thermistor (Apogee Instruments Inc., model ST-100). Vapor pressure deficit (VPD) was 1.25± 0.06/1.02 ± 0.08 kPa day/night measured with a relative humidity and temperature sensor (Campbell Scientific Inc., model HMP45A). Environmental measurements were made every ten seconds and ten-minute averages of the data were recorded with a datalogger (Campbell Scientific Inc., model CR1000X). Fans supplied airflow of about 0.8 m per s at the top of the canopy measured with a hot-wire anemometer (TSI Inc., model 8330)

### Nutrient solution composition

Nutrient solutions were mixed using deionized water and reagent grade salts (Sigma Aldrich). **Table 1** shows the composition of the nutrient solution. The concentration of elements followed mass balance principles as described by Bugbee (2004) and Langenfeld et al. (2022).

**Table 1:** The composition of the nutrient solution for the three P treatments.

P Treatment (mg L^-1^)	NH_4_-N	NO_3_-N	P	K	Ca	Mg	S	Si	Fe	Mn	Zn	B	Cu	Mo
	------------------------ mg L^-1^ --------------------------													
25			**25**	**143**										
			**(0.8)**	**(3.7)**										
50	25	103	**50**	**175**	60	20	26	8	1	0.2	0.4	0.4	1	0.01
	(1.8)	(7.4)	**(1.6)**	**(4.5)**	(1.5)	(0.8)	(0.8)	(0.3)	(18)	(3)	(3)	(40)	(16)	(0.1)
75			**75**	**208**										
			**(2.4)**	(5.3)										

Values in parentheses are in mM (macronutrients) or µM (micronutrients). The base solution was modified with monopotassium phosphate (KH_2_PO_4_) to supply 25, 50 or 75 mg per L P. Bold values indicate the final P and K concentration from KH_2_PO_4_. The pH was adjusted to 5.8 ± 0.2 using sulfuric acid (H_2_SO_4_). The EC of the solution was 1.41, 1.48 and 1.53 at 25, 50 and 75 mg per L, respectively.

### Irrigation and leachate monitoring

Plants were irrigated daily with 2 L per hour drip emitters (NetaFim) to about a 15% excess. Drippers were tested at the beginning and end of the study to ensure uniformity. Leachate from each tray was collected and monitored daily for pH and electrical conductivity (EC). pH measurements were made with an Oakton pHTestr 10 BNC pH meter. EC measurements were made with a Hanna Dist 4 EC meter. The average leachate pH over the study was 5.4 ± 0.3 and the average leachate EC was 1.2 ± 0.6 mS cm^-1^ (mean ± sd) across all treatments and reps. At weekly intervals, leachate P was measured using a colorimeter (LaMotte model Smart 3). The low range phosphate method was followed, and solutions were manually diluted to get within the 0.0 to 3.0 ppm PO_4_ range (LaMotte method 3653-SC). Elemental P concentration was calculated by multiplying the measured PO_4_ concentration by 0.326. The 0.326 multiplier is the molecular weight ratio of P to PO_4_.

### Plant measurements

Plant height was recorded from the base of the stem to the tallest part of the plant at the beginning and end of the study. At harvest, plants were manually separated into flowers, leaves, and stems. The leaves that subtend the inflorescence (sugar leaves) were included in the flower fraction. Fresh weight was recorded, and plants were placed on well ventilated shelves at 25°C and 30% relative humidity. After 5 days, dry mass was recorded. The biomass at this temperature and humidity was between 12 and 14% moisture. Flower yield (g m^-2^) was calculated as the total mass of flower per tray divided by the canopy area. Harvest index (HI) was calculated as the ratio of flower mass to total above ground biomass.

### Tissue element analysis

Approximately 1 g (dry) of leaf and 3 g (dry) flower tissue was sampled from each plant at harvest and analyzed for nutrient content. For element analysis, tissue was oven-dried at 80 C for 48 hours prior to analysis. The tissues from multiple plants in each tray were homogenized into one sample. Fan-leaves and flowers (with sugar leaves) from the top of the plant were used for analysis. Stems and roots were not analyzed. Tissue samples were analyzed using inductively coupled plasma optical emission spectroscopy (ICP-OES) at the Utah State University Analytical Laboratory (USUAL). USUAL is an accredited member of The North American Proficiency Testing Program (NAPT; naptprogram.org). Phosphorus use efficiency (PUE) was calculated as the total mass of P measured in the flower and leaf tissue divided by the total amount of applied P. Total recovery of P was calculated as the sum of P in the flowers, leaves and leachate divided by the total amount of applied P.

### Cannabinoid extraction

Flower material was sampled from dry flower buds at the top of the plant. Three flower buds (about 3 g dry) were sampled per plant. The dry material was ground to a fine powder using a stainless-steel coffee grinder and stored in plastic bags at 4°C until analysis. Approximately 100 to 300 mg of dry material was sampled and extracted with reagent grade methanol (10 mL methanol per 100 mg tissue). This was then vortexed for one minute and sonicated for 15 minutes. The sample was then diluted by adding 100 μL cannabinoid solution to 900 μL of reagent grade methanol and moved to a high-performance liquid chromatograph (HPLC) for cannabinoid quantification.

### Cannabinoid analysis

Sample extracts were analyzed using a Shimadzu model LC-2040C 3D Plus (Shimadzu Corporation, Kyoto, Japan) reverse phase high-performance liquid chromatography (HPLC) equipped with diode array detector and a NexLeaf CBX for Potency, superficially porous particle (SPP) 2.7 μm C18 4.6x 150 mm column (Shimadzu Corporation, Kyoto, Japan). The mobile phase was 0.085% phosphoric acid in acetonitrile (E_1_) and 0.085% phosphoric acid in distilled water (E_2_). Elution was performed by the following gradient: t_0 min_ = 70% (vv^-1^) E_2_; t_3 min_ = 70% (vv^-1^) E_2_; t_8 min_ = 85% (vv^-1^) E_2_; t_10 min =_ 95% (vv^- 1^) E_2_; t_11.01 min_ =70% (vv^-1^) E_2_. The flow rate was 1.6 mL per min. A reference standard containing the cannabinoid compounds of interest (Cayman Chemical Inc., Phytocannabinoid Mixture 11 (CRM)) was used to prepare the external calibration standards. All compounds were analyzed at 220 nm. CBDA had a retention time of 3.262 min; CBD had a retention time of 3.901 min; THCA had a retention time of 7.604 min; Δ9-THC had a retention time of 6.427 min; CBGA had a retention time of 3.519 min; and CBG had a retention time of 3.726 min. Minor cannabinoids were excluded from the analysis due to low concentrations. CBD, THC, and CBG equivalents (CBD_eq_, THC_eq_, and CBG_eq_) were calculated as described by Westmoreland et al. (2021).

### Statistical analysis

The study was a completely randomized design with three treatment levels (P input) and two replicates at each level (n=2). Each experimental unit consisted of three plants in three pots that shared a common tray for leachate collection. Flower yield was calculated as the sum of the dry flower from each tray divided by the growth area. For cannabinoids, each plant was sampled individually and the average of the three plants within an experimental unit was used for statistical analysis. For tissue element analysis, a representative sample of tissue from each plant was homogenized and analyzed as a single sample. Data were fit with a simple linear model in RStudio (R statistical software, version 4.1.0). Effects were considered significant at α = 0.05.

## Results

### Yield

All plants were green and healthy throughout the lifecycle, and, in spite of frequent close inspections, there were no visual differences among treatments at any time. Dry flower yield was unaffected by P input (**Supplementary Figure 1**; p = 0.20). The dry flower yield was 649 ± 41.5, (mean ± SD) grams per square meter (g m^-2^) averaged across all treatments. This resulted in a photon conversion efficacy of 0.26 ± 0.02 grams per mole of photons. PCE was calculated including photons from both the vegetative and flowering stage. Harvest index (HI) was 65 ± 0.02% averaged across all treatments. There was no effect of P input on HI (data not shown; p = 0.90).

### Cannabinoid concentration

We found no significant effect of P input on CBD_eq_ (p = 0.35), THC_eq_ (p = 0.38) or CBG_eq_ (p = 0.94) across P concentrations from 25 to 75 mg per L (**Supplementary Figure 2**). CBD_eq_ was 13.6 ± 0.58; THC_eq_ was 0.57 ± 0.03; and CBG_eq_ was 0.51 ± 0.02 averaged across treatments. The ratio of CBD_eq_ to THC_eq_ was identical among treatments at 23.8 ± 0.05.

### Tissue nutrient concentration

**Figure 1:**
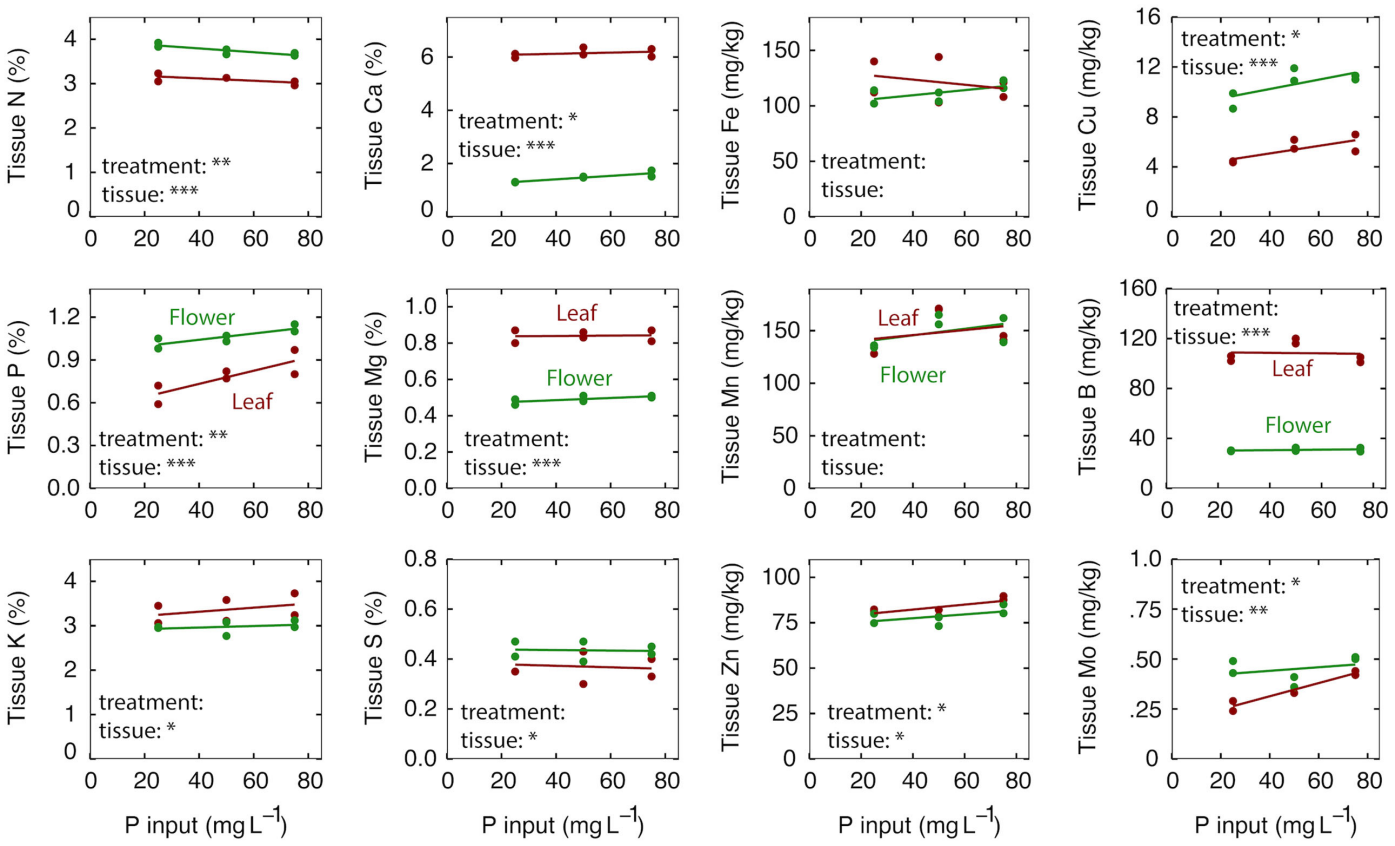
The effect of P input on tissue nutrient content at harvest after eight weeks of reproductive growth. Red lines represent leaf tissue; green lines represent flower tissue. Each point represents the average of plants in three separate containers that shared a common leachate collection tray. Significance codes for P treatment and tissue are as follows: *p < 0.05, **p < 0.01, ***p < 0.001. There was no interaction between P treatment and tissue for any element, so no significance codes are shown.

High rates of P can affect the uptake of other ions but increasing P supply resulted in only small differences in the nutrient content of flowers and leaves (**Figure 1**). The nutrient content of the leaves were within published optimal ranges for all P treatments (Cockson et al., 2019; Landis et al., 2019; Kalinowski et al., 2020).

For the macronutrients – N was reduced from 3.1 to 3.0% in the leaves and from 3.9 to 3.7% in the flowers as the P input increased from 25 to 75 mg per L (**Figure 1**; p = 0.04). As expected, P increased from 0.66 to 0.89% in the leaves and from 1.02 to 1.13% in the flowers as the P increased from 25 to 75 mg per L (**Figure 1**; p < 0.01). This translates to a 35% increase in the leaves and 11% increase in the flowers in response to a 3-fold increase in P input. Interestingly, K content in the leaves and flowers was unaffected by P treatment, despite a 45% increase in K (from KH_2_PO_4_) as the P input increased from 25 to 75 mg per L. Ca increased from 6.0 to 6.2% in the leaves and from 1.3 to 1.6% in the flowers with increasing P input (**Figure 1**; p = 0.05).

For the micronutrients – Cu increased from 4.9 to 5.9 mg kg^-1^ in the leaves and from 9.3 to 11.2 mg kg^-1^ in the flowers (p = 0.02); Zn increased from 82 to 89 mg kg^-1^ in the leaves and from 77 to 83 mg kg^-1^ in the flowers (p = 0.04); and Mo increased from 0.27 to 0.43 mg kg^-1^ in the leaves and from 0.46 to 0.51 mg kg^-1^ in the flowers (p = 0.01) as P increased from 25 to 75 mg per L (**Figure 1**).

### Nutrient partitioning between leaves and flowers

Nutrient content tended to be higher in the flowers than leaves for the mobile elements N, P and K, and higher in leaves than flowers for non-mobile elements Ca, B and Mg (**Figure 1**). Fe and Mn were the only elements that were not significantly different between leaves and flowers. Cu was twice as high in the flowers as in the leaves (**Table 2**). There was no interaction between P treatment and tissue type for any element tested (**Figure 1**).

**Table 2:** The ratio of nutrient concentration of flowers to leaves at harvest (data from **Figure 1**).

Flower to leaf ratio for tissue elements												
P Treatment (mg L^-1^)	N	P	K	Ca	Mg	S	Fe	Mn	Zn	B	Cu	Mo
Average	1.2**	1.4*	0.9	0.2***	0.6**	1.2**	0.9	1.0	0.9	0.3***	2.0**	1.3

There was no interaction between P treatment and the flower to leaf ratio, so the averages are presented. Values that are significantly different than 1 according to a t-test are denoted with an asterisk. Significance codes are as follows: * p <0.05, **p<0.01, ***p<0.001. The lower values indicate low phloem mobility (e.g. Ca, Mg and B); values higher than one indicate higher phloem mobility (e.g. N, P, S and Mo).

### Leachate analysis and phosphorus use efficiency

**Figure 2:**
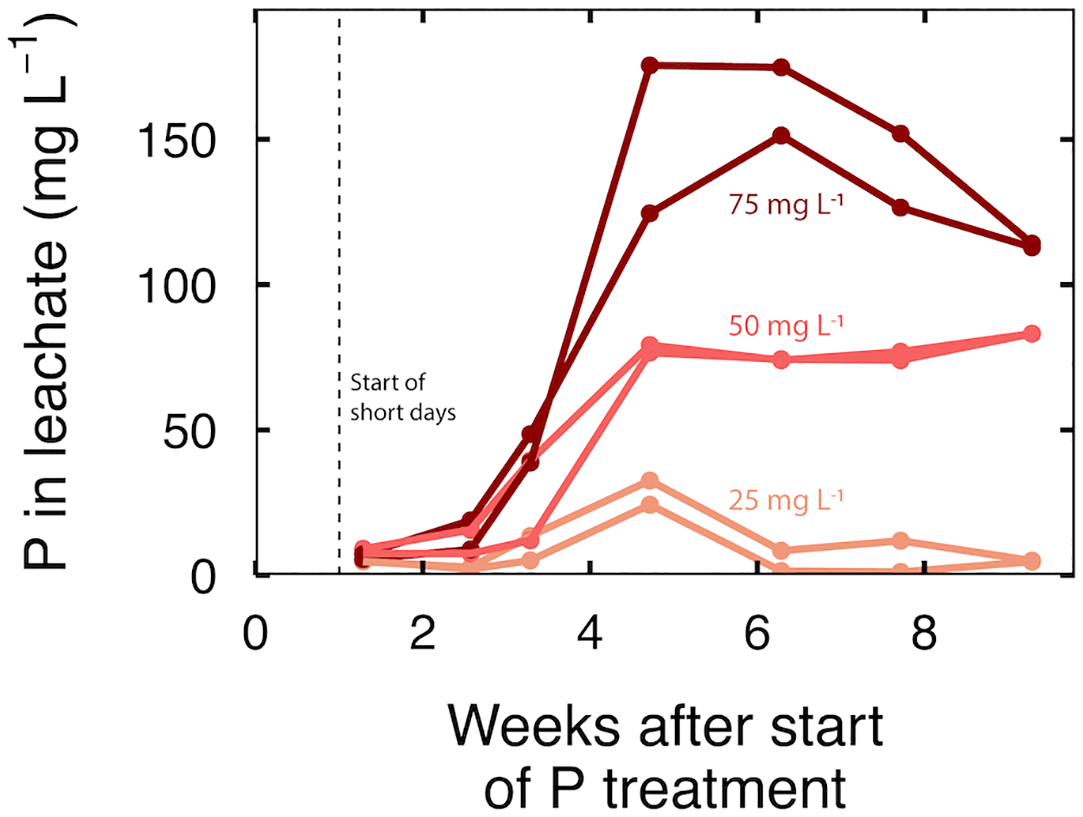
Time series of P in the leachate at an input P of 25, 50 and 75 mg per L. Each line represents a sample taken from a leachate collection tray that contained three separate containers.

P in the leachate increased to a maximum in all treatments at five weeks, followed by a decline or stable leachate P concentration for the final 4 weeks (**Figure 2**). As expected, increasing P input increased P in the leachate. At an input of 25 mg per L, leachate P concentration increased to a maximum of 28 mg per L, then dropped to less than 10 mg per L; at an input of 50 mg per L, P in the leachate increased to 75 mg per L and remained stable; at an input of 75 mg per L, P in the leachate increased to about 160 mg per L but declined to about 115 mg per L P at harvest (**Figure 2**).

**Figure 3:**
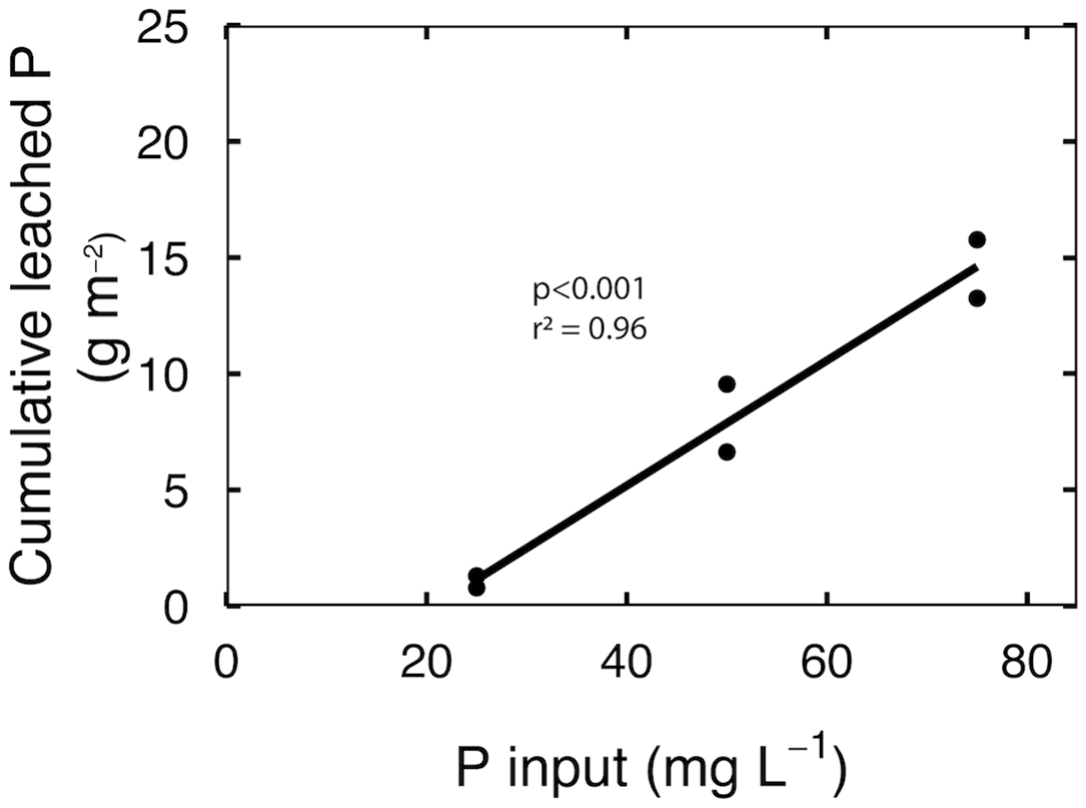
Cumulative leached P over eight weeks of reproductive growth at input P of 25, 50 and 75 mg per L. Each point represents the total leached P of a leachate collection tray that contained three separate containers.

The cumulative leached P was divided by the plant growth area to calculate grams of P leached per square meter. There was a linear increase in total leached P as input P increased from 25 to 75 mg per L P (**Figure 3**; p<0.001). Cumulative leached P increased by 5% for every 1 mg per L increase in input P, which translates to 12-fold increase in cumulative leached P in response to a 3-fold increase in input P.

**Figure 4:**
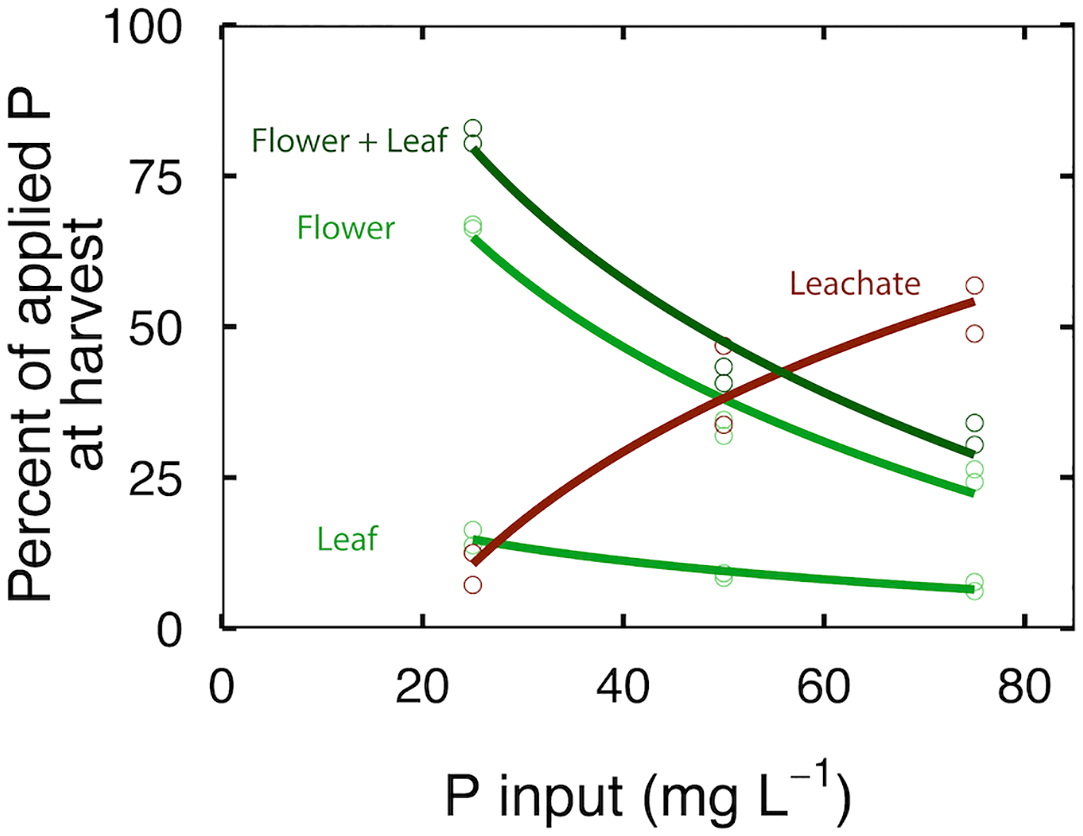
The effect of P input on recovery of P in flowers, leaves, and leachate. Each point represents plants in three separate containers that shared a common leachate collection tray. Curves represent second order polynomial functions fitted to the data.

At 25 mg per L, 65% of the applied P was in the flowers, 15% was in the leaves and 10% was in the leachate (**Figure 4**); the PUE was 80% and the total recovery of P was 91 ± 5.5%. At 50 mg per L, 35% of the applied P was in the flowers, 8% was in the leaves and 35% was in the leachate (**Figure 4**); the PUE was 41% and the total recovery of P was 82 ± 7.4%. At 75 mg per L, 25% of the P was in the flowers, 6% was in the leaves and 50% was in the leachate (**Figure 4**); the PUE was 31% and the total recovery of P was 85 ± 8.2%. The unrecovered P may have been in roots and stems, which were not analyzed.

## Discussion

The effect of phosphorus on growth, development and quality of *Cannabis* has been well studied over the recent years (Aubin et al., 2015; Bernstein et al., 2019; Cockson et al., 2020; Shiponi and Bernstein, 2021a; Shiponi and Bernstein, 2021b; Veazie et al., 2021), but few studies have examined nutrient partitioning between leaves and flowers and no studies have quantified the waste associated with over-fertilization.

Our data indicate that a P supply of 25 mg per L in continuous liquid feed was sufficient for maximum yield and cannabinoid concentration. This is generally consistent with Shiponi and Bernstein (2021b) who found no benefit of P above 30 mg per L in one high-THC cultivar. In contrast, Cockson et al. (2020) reported no additional benefit in yield or cannabinoid concentration above about 11 mg P per L. This low optimum for P could be caused by an increase in the volume of irrigation, which would increase the total P delivered to the root-zone. There could also be genetic variability in P requirements among cultivars (Cong et al., 2020).

Elevated root-zone P can cause iron deficiency and potentially reduce yield (Parry and Bugbee, 2017), but this was not observed in this study. This lack of an inhibitory effect of P on yield in Cannabis is consistent with multiple studies that have found no yield reduction between 25 and 75 mg P per L (Caplan et al., 2017a; Bernstein et al., 2019; Bevan et al., 2021; Shiponi and Bernstein, 2021b; Veazie et al., 2021). P toxicity is uncommon in crop plants, and there is variability in susceptibility among plant taxa (Lambers, 2022). P toxicity is typically caused by high root-zone P and an inability to regulate P transport at the root surface or store excess cytosolic P in vacuole (Han et al., 2022). *Cannabis* appears to tolerate elevated P without detrimental effects, but this is not a reason to over fertilize.

Most medical *Cannabis* is grown at elevated CO_2_, which closes stomates and thus changes the irrigation and fertilization demands. Water use efficiency (WUE) is the ratio of carbon fixed through photosynthesis to water lost through transpiration. At ambient CO_2_ (415 ppm), WUE is typically around 3 grams per L (Bugbee, 2004; Langenfeld et al., 2022). Using a mass balance approach, assuming an optimal leaf P concentration of 0.4% (Cockson et al., 2019; Landis et al., 2019; Kalinowski et al., 2020), the theoretical P demand is 12 mg per L at ambient CO_2_. Elevated CO_2_ reduces transpiration rate and increases photosynthetic rate (Morison, 1985). For this reason, WUE at elevated CO_2_ can be as high as 6 g per L (Bugbee, 2004; Langenfeld et al., 2022). Under these conditions, the theoretical P demand is 24 mg per L. It is important to consider other environmental factors, such as CO_2_, when formulating an optimal nutrient solution.

### Life-stage affects nutrient requirements

Recent studies indicate that P in continuous liquid feed is adequate at about 15 mg per L for vegetative growth in greenhouse conditions with ambient CO_2_ (Cockson et al., 2020; Veazie et al., 2021), but many studies, including this one, indicate higher levels of P are necessary for optimal reproductive growth (Bevan et al., 2021; Shiponi and Bernstein, 2021b). P uptake during reproductive growth may be much higher than during vegetative growth because developing inflorescences are enriched with P (**Figure 1**) and are a significant fraction of the biomass (Westmoreland et al., 2021). Although these inflorescences are a significant reservoir of P, this accumulation of P does not appear to be beneficial for yield or quality. This indicates that *Cannabis* has luxury uptake of P.

In medical *Cannabis* cultivation, growers actively prevent pollination and seed formation. This is significant because seeds of *Cannabis* contain high concentrations of storage P as phytic acid (PA) (Lott et al., 2000). PA is a critical source of P for developing seedlings in low-P soils, but it reduces the pool of active P in the plant and is unnecessary in medical *Cannabis*. The P in the flowers may be in the form of PA, but that is beyond the scope of this paper. Nevertheless, the high concentrations of P and PA in *Cannabis* seeds may explain the high uptake of P in medical *Cannabis* flowers.

Collectively, these data suggest a potential benefit of phasic P fertilization, where P supply is kept low during early growth and increased during late flowering to optimize yield (Correll, 1998). Future studies should examine increased P at different stages of reproductive growth.

### Genetic variability and the potential for selective breeding

Shiponi and Bernstein (2021b) studied two cultivars: a type I (THC dominant) and type II (roughly equal CBD and THC). There was no yield increase above 30 mg per L in the type I variety, but the yield of the type II variety increased by 30% from 30 to 90 mg per L. This interaction suggests the potential to genetically select *Cannabis* cultivars with a lower P requirement. Luxury uptake of K in fiber hemp has been reported (Finnan and Burke, 2013), but this was not observed in this study (**Figure 3**). This suggests there is the potential to select cultivars with less unproductive P in the flowers due to lower rates of luxury uptake.

We studied one cultivar in this study so it is difficult to draw conclusions about genetic variability in P uptake among *Cannabis* cultivars, but there is evidence from other crops to suggest potential differences that can be exploited through breeding. Cultivars of several crops vary in the accumulation of P. In groundnut, P in the seed ranged from 59 to 103 mg per 100 g, while PA-P ranged from 149 to 315 mg per 100 g (Hande et al., 2013). In soybean, P in seed ranged from 0.19 to 0.37 g per kg and PA-P ranged from 3.8 to 5.1 g per kg (Israel et al., 2006). In potato, the cultivar Pamella achieved maximum yield with no additional P application, whereas Desiree required 100 kg P_2_O_5_ per ha to achieve maximum yield (Daoui et al., 2014).

There are also differences in PUE among cultivars of crop plants in the field (Horst et al., 1993; Ciarelli et al., 1998; Rose et al., 2007; Powers et al., 2020; Beroueg et al., 2021). Differences in root architecture is a major contributor to this variability, with some cultivars more capable of increasing absorptive surface area under low P conditions. (Shenoy and Kalagudi, 2005; Beroueg et al., 2021). Cultivars can also vary in their ability to modify the rhizosphere by releasing organic acids and increasing the solubility of P in calcareous soils (Shenoy and Kalagudi, 2005), but this is not typically beneficial in controlled environment agriculture where adequate fertilizer is continuously supplied and substrate pH is tightly controlled.

### The environmental impact of *Cannabis* cultivation

*Cannabis* is becoming increasingly legal (Eastwood, 2020), leading to a rapid increase in cultivation. In northern California, the total area under cultivation increased by over 90% between 2012 and 2016 (Butsic et al., 2018). The environmental impact associated with this rapid increase is coming to the attention of cultivators, scientists and policy-makers (Mills, 2012; Wang et al., 2019; Summers et al., 2021; Zheng et al., 2021).

The effect of phosphorus on the environment has been well known for decades (Edwards and Harrold, 1970; Correll, 1998). *Cannabis* cultivation has been largely unregulated, but as legal cultivation continues to increase, there is a need to address the impact of over-application of fertilizer, especially P. Commercial cultivators can capitalize on the use of low P by marketing their product as sustainably grown.

There is a need for evidence-based recommendations for fertilization approaches that optimize *Cannabis* yield and quality with a focus on the associated environmental impact. In this study, an input P of 25 mg per L was sufficient for maximum growth and quality. The flowers accumulated high concentrations of P, but this did not result in higher flower yield or cannabinoid concentration.

Finally, we report a 12-fold increase in leached P in response to a 3-fold increase in input P. This study adds to the growing body of evidence indicating *Cannabis* does not benefit from excessive P fertilization and provides new insight into the accumulation and distribution among organs within the plant. Future studies should address genetic variation in P accumulation among *Cannabis* varieties and breeders should select for cultivars that accumulate less P in the flowers.

Controlled environment agriculture has the potential to reduce fertilizer and water use, but more research on precision nutrition is necessary to achieve this goal. Growers in field agriculture have been gradually adopting to strict regulations on fertilizer use; growers in controlled environments can set a new standard for sustainability for all types of crop production.

## Data availability statement

The original contributions presented in the study are included in the article. Further inquiries can be directed to the corresponding author.

## Author contributions

Conceptualization, FW and BB. Methodology, BB and FW. Formal analysis, FW. Investigation, FW. Writing—original draft preparation, FW. Writing—review and editing, FW and BB. Supervision, BB. Funding acquisition, BB. All authors contributed to the article and approved the submitted version.

## Funding

This study received funding from Utah Agricultural Experiment Station, SunMed Growers, METER Group Inc. and Harborside. The funders were not involved in the study design, collection, analysis, interpretation of data, the writing of this article or the decision to submit it for publication. Approved as UAES Journal Paper Number 9598.

## Acknowledgments

We thank Alec Hay for technical assistance, Julie Hershkowitz for assistance with data collection, and Dr. Casey Simons for cannabinoid analysis. We also thank the Utah Department of Agriculture and Food for providing a certificate to research industrial hemp.

## Conflict of interest

The authors declare that the research was conducted in the absence of any commercial or financial relationships that could be construed as a potential conflict of interest.

This study received funding from SunMed Growers, METER Group Inc. and Harborside. The funders were not involved in the study design, collection, analysis, interpretation of data, the writing of this article or the decision to submit it for publication.

## Publisher’s note

All claims expressed in this article are solely those of the authors and do not necessarily represent those of their affiliated organizations, or those of the publisher, the editors and the reviewers. Any product that may be evaluated in this article, or claim that may be made by its manufacturer, is not guaranteed or endorsed by the publisher.
