# Supplementary material for: Sustainable Cannabis Nutrition: Elevated root-zone phosphorus significantly increases leachate P and does not improve yield or quality
Source: Front Plant Sci. 2022 Nov 17;13:1015652. doi: 10.3389/fpls.2022.1015652 (PMC9724152; doi:10.3389/fpls.2022.1015652)
Supplement: Supplementary file 1 [file DataSheet_1.pdf]

Supplementary Material

1.1 Supplementary Figures

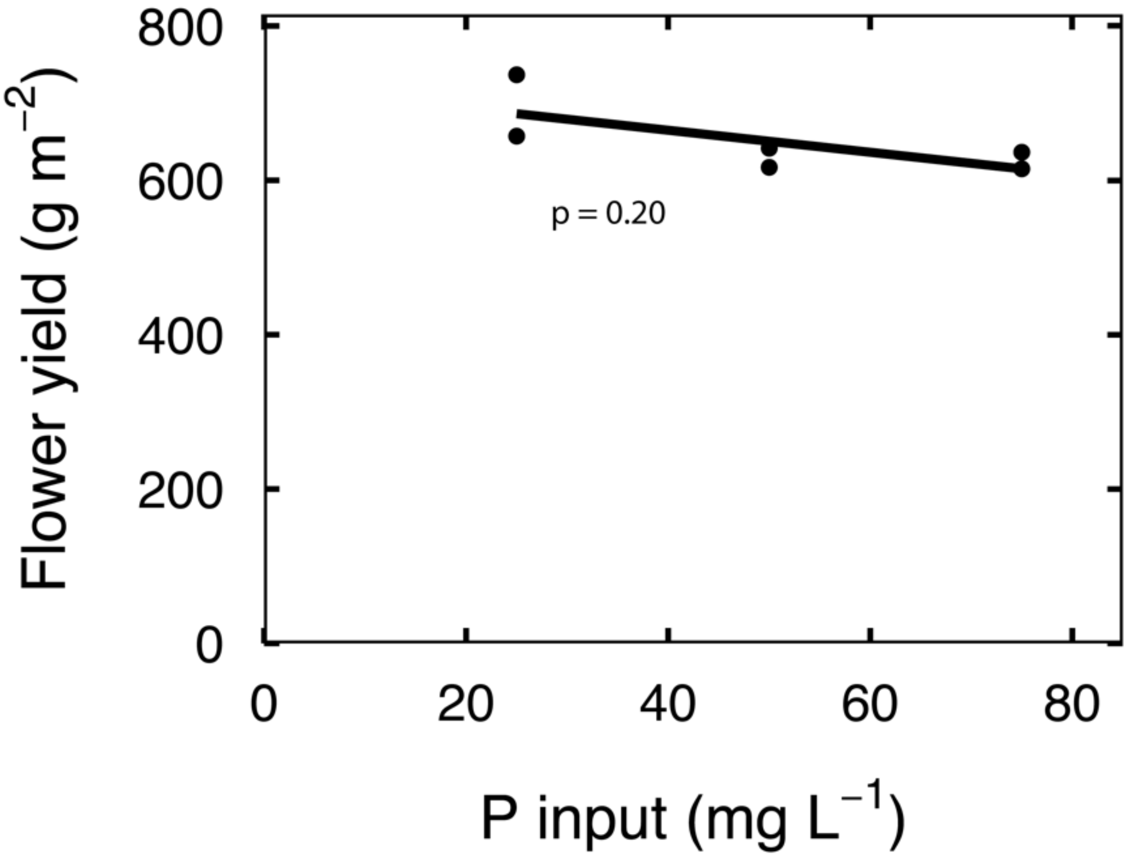

Supplementary Figure 1. The effect of P input on dry flower yield at harvest after eight weeks of reproductive growth. Each point represents the yield of plants in three separate containers that shared a com-mon leachate collection tray. There was no statistically significant effect of P input on flower yield ($p = 0.20$)

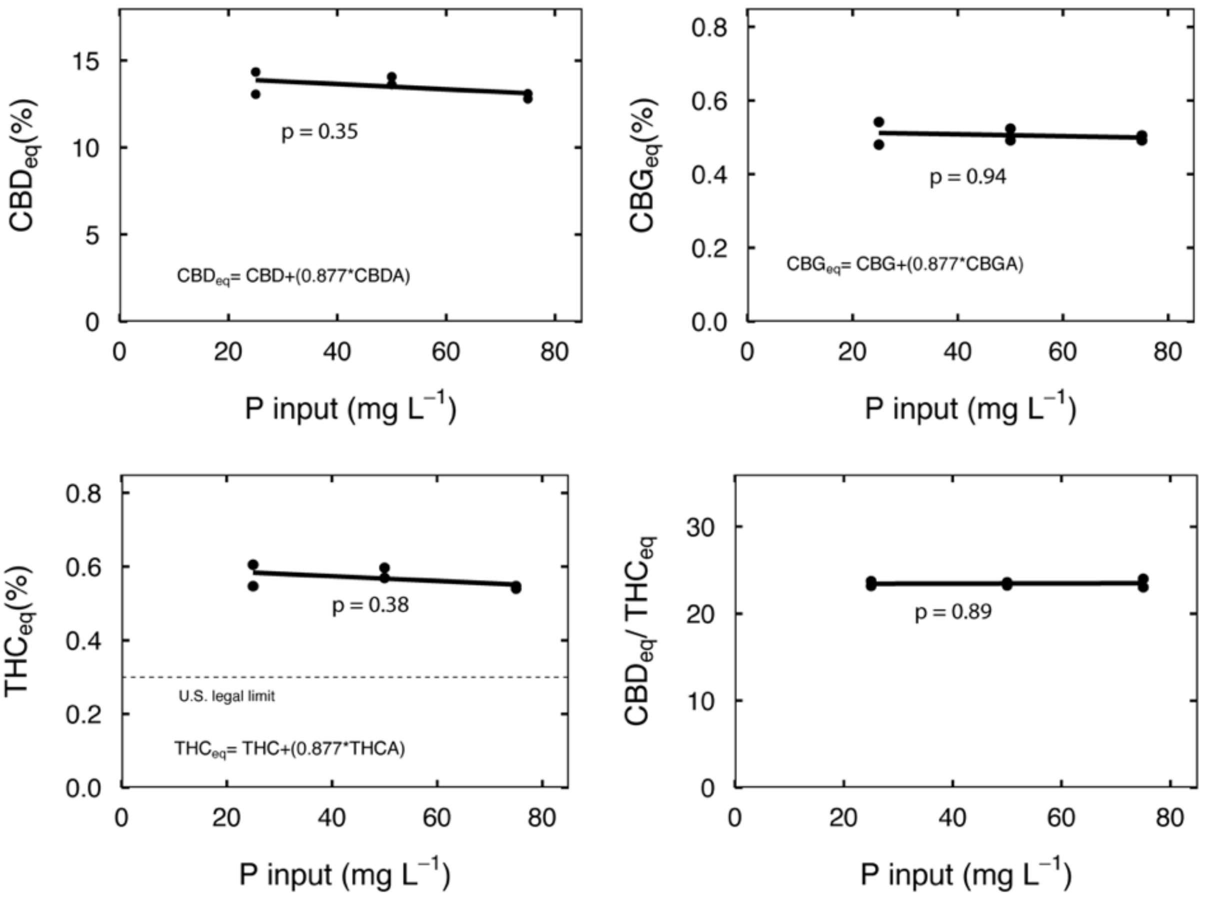

Supplementary Figure 2. The effect of P input on CBDeq, THCeq, CBGeq concentration and CBDeq/THCeq ratio in dry flower material at harvest after eight weeks of reproductive growth. Each point represents the average of plants in three separate containers that shared a common leachate collection tray. There was no effect of P input on any of the cannabinoids tested. The U.S. legal limit for THC_{eq} in hemp is 0.3%.
